# Efficacy observation and prognosis analysis of EGFR-TKIs alone versus EGFR-TKIs plus chemotherapy in advanced lung adenocarcinoma with EGFR Exon 19 Deletion, Exon 21 L858R mutation: A historical cohort study

**DOI:** 10.1097/MD.0000000000034110

**Published:** 2023-06-30

**Authors:** Jinhua Zhou, Hongya Qin, Jianlong Miao, Ruijuan Liu, Wei Wang

**Affiliations:** a Department of Respiratory Medicine, The Second Hospital of Shandong University, Jinan, P.R. China; b Department of Respiratory Medicine, Jining First People’s Hospital, Jining, P.R. China.

**Keywords:** chemotherapy, efficacy, EGFR-TKI, lung adenocarcinoma, prognosis

## Abstract

The aim of this study was to investigate the clinical efficacy and determine the prognostic value of Epidermal growth factor receptor-tyrosine kinase inhibitor (EGFR-TKIs) alone versus EGFR-TKIs plus chemotherapy for the treatment of advanced lung adenocarcinoma with EGFR Exon 19 Deletion(19Del), Exon 21 L858R (L858R) mutation. The demographic and clinical characteristics of 110 newly diagnosed metastatic lung adenocarcinoma patients with the EGFR 19Del, L858R mutation from June 2016 to October 2018 were retrospectively analyzed. Total remission rate (ORR), disease control rate (DCR), median progression-free survival (mPFS), and patient 1-year/2-year survival between EGFR-TKIs combined with first-line platinum-containing double-drug chemotherapy (Observation) group and an EGFR-TKIs alone (Control) group were evaluated and analyzed. For lung adenocarcinoma patients with the EGFR 19Del, L858R mutation, the Observation group had a better ORR (81.4% vs 52.2%), mPFS (12.0 vs 9 months), and 2-year survival (72.1% vs 52.2%) than the Control group, and the differences were statistically significant (*P <* .05), but DCR (95.3% vs 88.1%) and 1-year survival (90.7% vs 83.6%) were not significantly different between the groups (*P* > .05). For lung adenocarcinoma with the EGFR 19Del mutation, the Observation group showed a better ORR (81.8% vs 54.3%), and mPFS (14.5 vs 11.0 months) than the Control group, and the differences were statistically significant (*P <* .05), but DCR (95.5% vs 91.4%), 1-year survival (90.9% vs 85.7%), and 2-year survival (72.7% vs 60.0%) were not significantly different (*P >* .05). For lung adenocarcinoma with the EGFR L858R mutation, the Observation group showed a better ORR (81.0% vs 50.0%), mPFS (12.0 vs 9.0 months), and 2-year survival (71.4% vs 43.8%) than the Control group (*P <* .05), but DCR (95.2% vs 84.4%) and 1-year survival (90.5% vs 81.3%) were not significantly different (*P >* .05). Compared to EGFR-TKIs alone, EGFR-TKIs combined with chemotherapy improved ORR and mPFS in cases of advanced lung adenocarcinoma with EGFR 19Del, L858R mutation. In particular, patients with the EGFR L858R mutation showed a long-term survival benefit trend. EGFR-TKIs combined chemotherapy may therefore be a viable treatment method for delaying targeted drug resistance.

## 1. Introduction

Lung cancer is a disease with a global high incidence and mortality rate, that affects human health in serious ways. The number of patients diagnosed with advanced lung cancer continues to increase every year. Lung adenocarcinoma is the most common pathological type of lung cancer, accounting for about 40% of total lung cancer cases.^[1–3]^ Therefore, the treatment and management of advanced lung adenocarcinoma is of particular importance. Over the past decade, many studies have demonstrated that first-line epidermal growth factor receptor tyrosine kinase inhibitor (EFGR-TKI) therapy for patients with advanced lung adenocarcinoma, who were selected on the basis of epidermal growth factor receptor (EGFR) mutations, significantly improved progression-free survival.^[4–10]^ Although significant progress has been made in targeted treatment for lung cancer, the dream of curing driver-positive lung adenocarcinoma, or even downgrading it to a chronic disease and achieving long-term remission, remains unrealized due to drug resistance issues. One strategy to delay or reduce targeted drug resistance is to combine targeted drugs with chemotherapy. Studies such as NEJ009 and NCT02148380 suggested that EGFR-TKIs combined with chemotherapy could provide additional survival benefits for patients,^[11,12]^ but the clinical efficacy of combined therapy on different EGFR mutant subtypes is still controversial and needs further verification. This study therefore aimed to investigate the clinical efficacy and prognosis traits of EGFR-TKIs alone versus EGFR-TKIs plus chemotherapy for advanced lung adenocarcinoma with EGFR 19Del, L858R mutation.

## 2. Methods

### 2.1. Patients and treatments

This was a historical cohort study. All data were collected from outpatient ward medical records in the system or via telephone follow-ups, no treatment intervention. The study conformed to the principles of the Declaration of Helsinki and was approved by the ethics review committee of the Jining First People’s Hospital (approval number: 2020-035) and the requirement for informed consent was waived.

The main eligibility criteria for patients were as follows: a diagnosis of stage IIIB (all patients have refused curative chemoradiotherapy for personal reasons) or IV lung adenocarcinoma with EGFR mutation (exon 19Del, exon 21 L858R), age of 18 to 75 years, Eastern Cooperative Oncology Group performance status (PS) of 0 to 1 and adequate organ function, and at least 1 measurable lesion in any area except the skull, according to RECIST 1.1 criteria. The main exclusion criteria for patients were as follows: cancer accompanied by brain metastasis or cerebral membrane metastasis, those who had previously undergone other forms of anti-tumor treatments, serious concomitant systemic disorders, interstitial pneumonia, another primary malignancy, preexisting T790M mutation, and symptomatic brain metastases.

Eligible patients were divided into EGFR-TKIs plus chemotherapy (Observation group) and EGFR-TKIs alone (Control group) based on their treatment. Observation group: gefitinib 250 mg orally once per day or icotinib orally 3 times per day, plus first-line platinum-containing double-drug chemotherapy in a 3-week cycle for 4 cycles; after chemotherapy patients who did not experience cancer progression were maintained in oral targeted drugs, joint or non-joint pemetrexed single-agent chemotherapy; Control group: gefitinib 250 mg orally once per day or icotinib orally 3 times per day taken continuously. The short- and long-term efficacies of these regimens on patients with EGFR 19Del or L858R mutation were observed.

### 2.2. Clinical assessments and study endpoints

According to RECIST 1.1 criteria, the Observation group received a comprehensive examination every 2 chemotherapy cycles. The Control group received a comprehensive examination every 2 months, after being evaluated as effective or stable during the first month. The examination items include chest enhanced computed tomography (CT), abdominal B-ultrasound or enhanced CT, tumor markers, regular reexamination of Magnetic Resonance (MR) or bone scan for patients with bone metastases.

Treatment efficacy was evaluated for all patients after 3 months, including parameters, such as complete response (CR), partial response (PR), disease progression (DP), and stable condition. Objective response rate (ORR) was calculated as ORR = CR + PR/total number of cases × 100%, and disease control rate (DCR) was calculated as DCR = CR + PR + stable condition/total number of cases × 100%. Progression-free survival (PFS) was defined as the time from the start of treatment to disease progression or death from any cause. Patient survival was recorded from the start of treatment until 1 and 2 years post-treatment. The primary study endpoint was median progression-free survival (mPFS) and the secondary endpoints were ORR, DCR, and 1- and 2-year survival rates.

### 2.3. Statistical analysis

Clinical characteristics, ORR, DCR, and 1- and 2-year survival rates were measured by Chi-square test or Fisher’s exact test, and the classification variables were expressed as percentages. The Kaplan–Meier method was used to draw survival curves for mPFS survival data, and the log-rank method was used to compare whether there were differences in survival distribution curves. *P* < .05 was considered significant.

## 3. Results

### 3.1. Patient characteristics

A total of 110 patients were included in this study, including 43 in the observation group (22 patients with the 19Del mutation and 21 patients with the L858R mutation) and 67 (35 patients with the 19Del mutation and 32 patients with the L858R mutation) in the control group. The characteristics of patients were well balanced between the groups (Tables 1 and 2).

**Table 1:** Clinical characteristics of patients with EGFR 19Del, L858R mutation (n, %).

Clinical characteristics	EGFR 19Del mutation (n = 57)	EGFR L858R mutation (n = 53)	χ^2^	*P*
Sex				
Male	24 (42.1%)	19 (35.8%)	0.451	.502
Female	33 (57.9%)	34 (64.2%)		
Age, yr				
≤60	27 (47.4%)	19 (35.8%)	1.498	.221
>60	30 (52.6%)	34 (64.2%)		
Smoking status				
Never	36 (63.2%)	38 (71.7%)	0.910	.340
Previous or current smoker	21 (36.8%)	15 (28.3%)		
Family history of cancer				
No	40 (70.2%)	38 (71.7%)	0.031	.861
Yes	17 (29.8%)	15 (28.3%)		
Lung disease history				
No	40 (70.2%)	40 (75.5%)	0.388	.533
Yes	17 (29.8%)	13 (24.5%)		
Clinical stage				
IIIB	12 (21.1%)	7 (13.2%)	1.183	.277
IV	45 (78.9%)	46 (86.8%)		
ECOG-PS				
0	37 (64.9%)	32 (60.4%)	0.242	.623
1–2	20 (35.1%)	21 (39.6%)		
Distant transition number				
0–1	34 (59.7%)	29 (54.7%)	0.273	.601
≥2	23 (40.3%)	24 (45.3%)		

ECOG-PS = eastern cooper oncology group-performance status, EGFR = epidermal growth factor receptor.

**Table 2:** Characteristics of patients in observation group and control group (n, %).

Clinical characteristics	Observation group (n = 43)	Control group (n = 67)	χ^2^	*P*
Sex				
Male	13 (30.2%)	30 (44.8%)	2.327	.127
Female	30 (69.8%)	37 (55.2%)		
Age, yr				
≤60	21 (48.8%)	25 (37.3%)	1.430	.232
>60	22 (52.63%)	42 (62.7%)		
Smoking status				
Never	29 (67.4%)	45 (67.2%)	0.001	.976
Previous or current smoker	14 (32.6%)	22 (32.8%)		
Family history of cancer				
No	29 (67.4%)	49 (73.1%)	0.411	.512
Yes	14 (32.6%)	18 (26.9%)		
Lung disease history				
No	33 (76.7%)	47 (70.1%)	0.574	.449
Yes	10 (23.3%)	20 (29.9%)		
Mutation				
19Del	22 (51.2%)	21 (48.8%)	0.012	.912
L858R	35 (52.2%)	32 (47.8%)		
Targeted drugs				
Gefitinib	20 (46.5%)	38 (56.7%)	1.094	.296
Icotinib	23 (53.5%)	29 (43.3%)		
Clinical stage				
IIIB	9 (20.9%)	10 (14.9%)	0.661	.416
IV	34 (79.1%)	57 (85.1%)		
ECOG-PS				
0	29 (67.4%)	40 (59.7%)	0.671	.413
1–2	14 (32.6%)	27 (40.3%)		
Distant transition number				
0–1	27 (62.8%)	36 (53.7%)	0.878	.349
≥2	16 (37.2%)	31 (46.3%)		

ECOG-PS = eastern cooper oncology group-performance status, EGFR = epidermal growth factor receptor.

### 3.2. Efficacy

**Figure 1.**
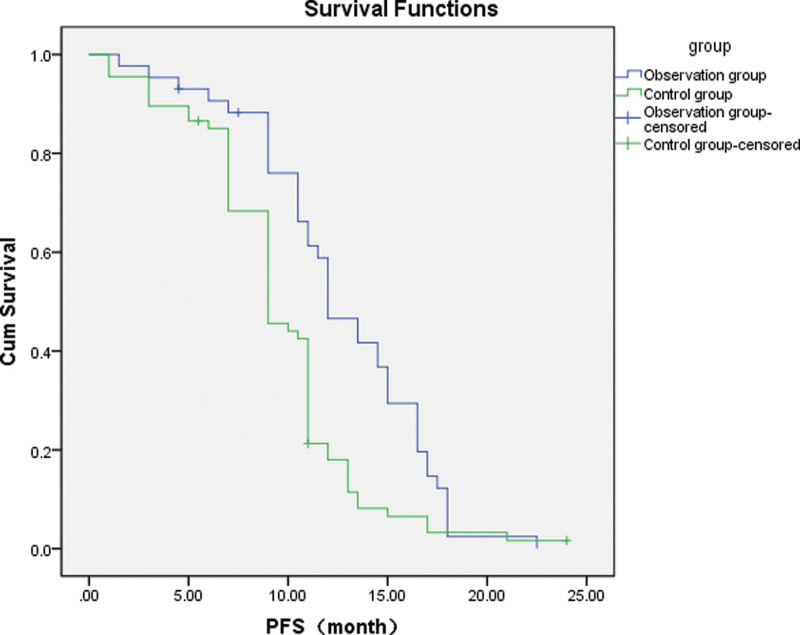
Survival curves of patients with EGFR 19Del or L858R mutations in the observation and control groups. The blue curve represents the mPFS of the observation group: 12.0 months (95% CI: 10.220–13.780 months), and the green curve represents the mPFS of the control group: 9.0 months (95% CI: 9.854–12.146 months). The difference between the groups was statistically significant (*P =* .001). EGFR = epidermal growth factor receptor, mPFS = median progression-free survival, PFS = progression-free survival.

**Figure 2.**
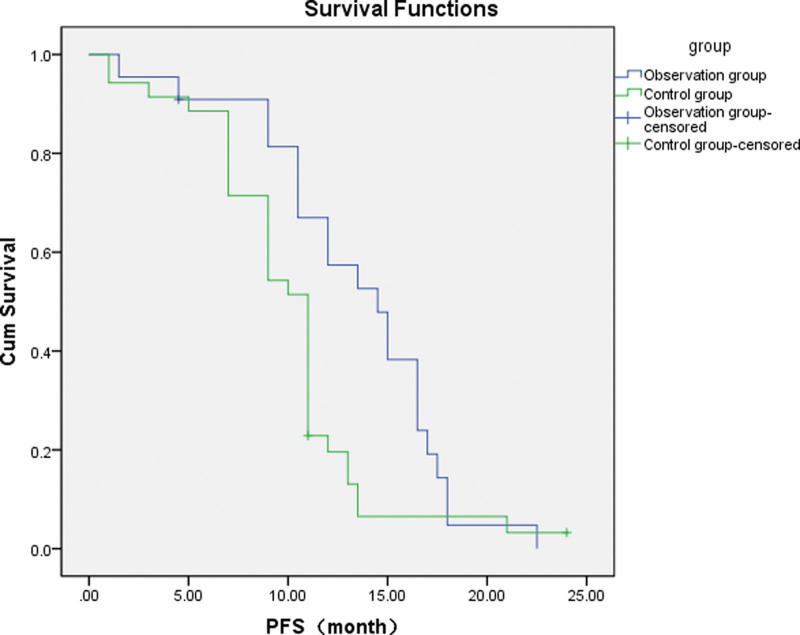
Survival curves of patients with the EGFR 19Del mutation in the observation and control groups. The blue curve represents the mPFS of the observation group (EGFR 19Del mutation): 14.5 months (95% CI: 11.151–17.849 months), and the green curve represents the mPFS of the control group (EGFR 19Del mutation): 11.0 months (95% CI: 9.854–12.146 months). The difference between the groups was statistically significant (*P =* .025). EGFR = epidermal growth factor receptor, mPFS = median progression-free survival, PFS = progression-free survival.

**Figure 3.**
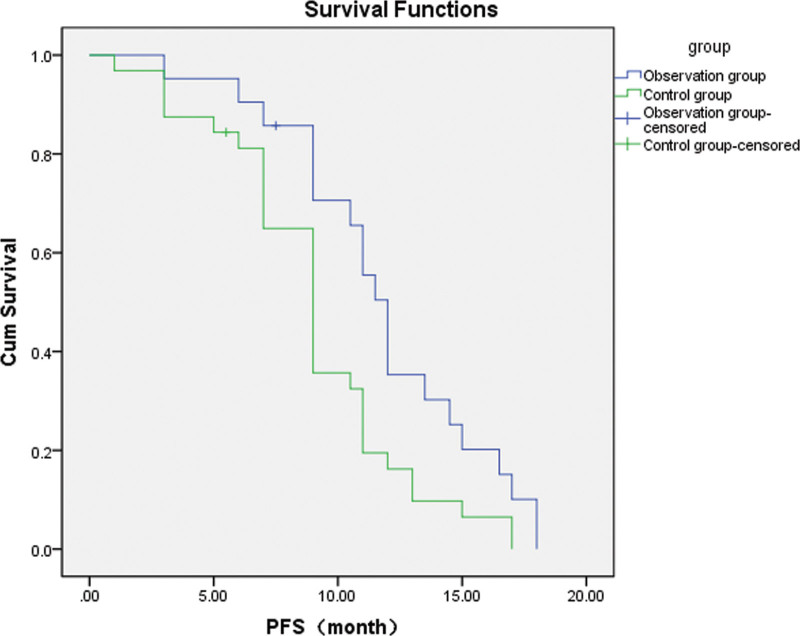
Survival curves of patients with the EGFR L858R mutation in the observation and control groups. The blue curve represents the mPFS of the observation group (EGFR L858R mutation): 12.0 months (95% CI: 10.960–13.040 months), and the green curve represents the mPFS of the control group (EGFR L858R mutation): 9.0 months (95% CI: 7.844–10.156 months). The difference between groups was statistically significant (*P =* .019). EGFR = epidermal growth factor receptor, mPFS = median progression-free survival, PFS = progression-free survival.

1. For lung adenocarcinoma with EGFR 19Del, L858R mutation, the Observation group showed a better ORR, mPFS and 2-year survival than the Control group. DCR and 1-year survival, however, were not significantly different (Tables 3 and 4; Fig. 1).
2. For lung adenocarcinoma with just EGFR 19Del mutation, the Observation group showed a better ORR and mPFS than the EGFR-TKIs alone group, but DCR and 1-year/2-year survival were not significantly different (Tables 5 and 6; Fig. 2).
3. For lung adenocarcinoma with just EGFR L858R mutation, the Observation group had a better ORR, mPFS, and 2-year survival than the EGFR-TKIs alone group, but DCR and 1-year survival were not significantly different (Tables 7 and 8; Fig. 3).

**Table 3:** Short-term efficacy of patients with EGFR 19Del, L858R mutation (n, %).

Efficacy	Observation group (n = 43)	Control group (n = 67)	χ^2^	*P*
CR	1	1	–	–
PR	34	34	–	–
SD	6	24	–	–
PD	2	8	–	–
ORR	18 (81.4%)	19 (52.2%)	9.622	.002
DCR	21 (95.3%)	32 (88.1%)	0.917	.338

CR = complete response, DCR = disease control rate, EGFR = epidermal growth factor receptor, ORR = objective response rate, PD = progressive disease, PR = partial response, SD = stable disease.

**Table 4:** Survival in patients with EGFR 19Del, L858R mutation (n, %).

Survival time	Observation group (n = 43)	Control group (n = 67)	χ^2^	*P*
1-year survival	39 (90.7%)	56 (83.6%)	1.126	.289
2-year survival	31 (72.1%)	35 (52.2%)	4.302	.038

EGFR = epidermal growth factor receptor.

**Table 5:** Short-term efficacy of just EGFR 19Del mutation (n, %).

Efficacy	Observation group (EGFR 19Del mutation) (n = 22)	Control group (EGFR 19Del mutation) (n = 35)	χ^2^	*P*
CR	1	1	–	–
PR	17	18	–	–
SD	3	13	–	–
PD	1	3	–	–
ORR	18 (81.8%)	19 (54.3%)	4.496	.034
DCR	21 (95.5%)	32 (91.4%)	0.002	.963

CR = complete response, DCR = disease control rate, EGFR = epidermal growth factor receptor, ORR = objective response rate, PD = progressive disease, PR = partial response, SD = stable disease.

**Table 6:** Survival in patients with just EGFR 19Del mutation (n, %).

Survival time	Observation group (EGFR 19Del mutation)(n = 22)	Control group (EGFR 19Del mutation) (n = 35)	χ^2^	*P*
1-year survival	20 (90.9%)	30 (85.7%)	0.028	.867
2-year survival	16 (72.7%)	21 (60.0%)	0.961	.327

EGFR = epidermal growth factor receptor.

**Table 7:** Short-term efficacy of just EGFR L858R mutation (n, %).

Efficacy	Observation group (EGFR L858R mutation) (n = 21)	Control group (EGFR L858R mutation) (n = 32)	χ^2^	*P*
CR	0	0	–	–
PR	17	16	–	–
SD	3	11	–	–
PD	1	5	–	–
ORR	17 (81.0%)	16 (50.0%)	5.170	.023
DCR	20 (95.2%)	27 (84.4%)	0.605	.437

CR = complete response, DCR = disease control rate, EGFR = epidermal growth factor receptor, ORR = objective response rate, PD = progressive disease, PR = partial response, SD = stable disease.

**Table 8:** Survival in patients with just EGFR L858R mutation (n, %).

Survival time	Observation group (EGFR L858R mutation) (n = 21)	Control group (EGFR 21-L858R mutation) (n = 32)	χ^2^	*P*
1-year survival	19 (90.5%)	26 (81.3%)	0.276	.599
2-year survival	15 (71.4%)	14 (43.8%)	3.920	.048

EGFR = epidermal growth factor receptor.

## 4. Discussion

Targeted drugs have brought lung cancer into the era of precision treatment, but they can often lead to drug resistance. The T790M mutation, detected in tissue specimens of about 50% to 60% of patients with acquired resistance, is considered to be the most common cause of resistance to the EGFR-TKI drug generation.^[13]^ How to avoid or delay the emergence of targeted drug resistance has become an urgent problem to be solved. NEJ009 is the first phase III study to evaluate the efficacy of a combination of EGFR-TKI plus platinum combination therapy, used in conjunction with standard EGFR-TKI monotherapy for the treatment of advanced non-small-cell lung cancer harboring EGFR mutations. One phase III study in India demonstrated a significant benefit of the combination regimen compared to gefitinib alone (mmPFS, 16 vs 8 months; hazard ratio, HR 0.51).^[14]^ This means the combination regimen delayed the development of resistance.

Targeted combination chemotherapy may be an effective treatment model to delay purely-targeted drug resistance, and some studies believe that prolonged mPFS and long-term survival data for patients who have undergone this type of therapy have not yet been clearly defined. At present, the mechanism of targeted therapy combined with chemotherapy to improve efficacy is not very clear. Some studies have shown that chemotherapy used before or at the same time as targeted therapy may induce EGFR expression in tumor cells and enhance signal transduction, thus improving the targeting and sensitivity of EGFR-TKI.^[15]^ Another study found that tumors with high EGFR expression were relatively sensitive to chemotherapy, whereas tumor resistance to chemotherapy was associated with its decreased growth signal.^[16]^ EGFR-TKI can effectively inhibit the repair of chemotherapy-induced DNA damage, maintain the intracellular concentration of chemotherapeutic agents, promote and maintain the chemotherapy-induced apoptosis of tumor cells, and affect the expression and secretion of vascular endothelial growth factor (VEGF), transforming growth factor alpha (TGF-α), and interleukin-8 (IL-8) in the tumor microenvironment, thus inhibiting the initial formation of tumor microvessels following chemotherapy.^[17]^

Although long-term survival data are lacking, targeted combination chemotherapy has certain advantages in delaying targeted drug resistance. Because of tumor heterogeneity, patients with different EGFR mutation sites have different sensitivities to targeted drugs. For EGFR mutation-positive patients included in previous studies of targeted combination treatment modes, it is rare to see further follow-ups regarding mutation sites, and treatment efficacy against different mutant subtypes is still controversial. In this study, the clinical characteristics of patients with advanced lung adenocarcinoma with EGFR 19Del, L858R mutation were retrospectively analyzed, and the efficacy of targeted combined with platinum two-drug chemotherapy was compared with that of targeted therapy alone. At the same time, the sensitivities of patients with mutations at different sites (19Del or L858R) to EGFR-TKI or combination platinum-containing two-drug chemotherapy were also investigated. Compared to EGFR-TKIs alone, EGFR-TKIs combined with chemotherapy improved ORR and mPFS in advanced lung adenocarcinoma with EGFR 19Del, L858R mutation. In particular, patients with EGFR L858R mutation showed a long-term survival benefit trend. The results of our study are consistent with those of other reports in the literature.^[12,18]^

This study did not assess drug safety related indicators. However, numerous studies have found that combination therapy compared to pure targeted therapy, although often higher in the incidence of adverse reactions, still shows a controllable tolerance overall.^[14,19]^ These types of reactions were retrospectively analyzed in this study, and we did not find any that were related to patient deaths. The efficacy and safety of EGFR-TKIs alone versus EGFR-TKIs plus chemotherapy for treatment of advanced lung adenocarcinoma with EGFR 19Del, L858R mutation still needs to be further investigated in large prospective clinical trials.

## Acknowledgments

My deepest gratitude to Professor Wang, Professor Liu, Hongya Qin and Jianlong Miao. Due to their constant encouragement and guidance, we finished this manuscript and solved problems during the difficult course of the manuscript with mutual help and joint efforts. All authors have read the manuscript and given permission to be named and they have no conflicts of interest to disclose. We would like to thank Editage (www.editage.cn) for English language editing.

## Author contributions

**Conceptualization:** Ruijuan Liu.

**Data curation:** Jinhua Zhou, Jianlong Miao.

**Investigation:** Hongya Qin.

**Methodology:** Jinhua Zhou, Ruijuan Liu, Wei Wang.

**Project administration:** Jianlong Miao.

**Supervision:** Wei Wang.

**Validation:** Ruijuan Liu.

**Visualization:** Wei Wang.

**Writing – original draft:** Jinhua Zhou.

**Writing – review & editing:** Hongya Qin.
